# Calycosin Alleviates Paraquat-Induced Neurodegeneration by Improving Mitochondrial Functions and Regulating Autophagy in a *Drosophila* Model of Parkinson’s Disease

**DOI:** 10.3390/antiox11020222

**Published:** 2022-01-24

**Authors:** Hitesh Singh Chaouhan, Xin Li, Kuo-Ting Sun, I-Kuan Wang, Tung-Min Yu, Shao-Hua Yu, Kuen-Bao Chen, Wei-Yong Lin, Chi-Yuan Li

**Affiliations:** 1 Graduate Institute of Biomedical Sciences, China Medical University, Taichung 40402, Taiwan; hschaouhan191986@gmail.com (H.S.C.); papa82988@gmail.com (X.L.); 2 Department of Pediatric Dentistry, China Medical University Hospital, Taichung 40402, Taiwan; duke111053@hotmail.com; 3 School of Dentistry, China Medical University, Taichung 40402, Taiwan; 4 Division of Nephrology, China Medical University Hospital, Taichung 40402, Taiwan; ikwang@seed.net.tw; 5 Department of Internal Medicine, School of Medicine, China Medical University, Taichung 40402, Taiwan; 6 Division of Nephrology, Department of Internal Medicine, Taichung Veterans General Hospital, Taichung 40402, Taiwan; yu5523@gmail.com; 7 School of Medicine, China Medical University, Taichung 40402, Taiwan; 8 Department of Emergency Medicine, China Medical University Hospital, Taichung 40402, Taiwan; shaoyu@mail.cmu.edu.tw; 9 Department of Anesthesiology, China Medical University Hospital, Taichung 40402, Taiwan; 10 Department of Medical Research, China Medical University Hospital, Taichung 40402, Taiwan; 11 Graduate Institute of Integrated Medicine, China Medical University, Taichung 40402, Taiwan; 12 Brain Diseases Research Center, China Medical University, Taichung 40402, Taiwan

**Keywords:** Parkinson’s disease, paraquat, calycosin, neurodegeneration, autophagy, *Drosophila*

## Abstract

Parkinson’s disease (PD) is the second most common age-related neurodegenerative disorder with limited clinical treatments. The occurrence of PD includes both genetic and environmental toxins, such as the pesticides paraquat (PQ), as major contributors to PD pathology in both invertebrate and mammalian models. Calycosin, an isoflavone phytoestrogen, has multiple pharmacological properties, including neuroprotective activity. However, the paucity of information regarding the neuroprotective potential of calycosin on PQ-induced neurodegeneration led us to explore whether calycosin can mitigate PD-like phenotypes and the underlying molecular mechanisms. We used a PQ-induced PD model in Drosophila as a cost-effective in vivo screening platform to investigate the neuroprotective efficacy of natural compounds on PD. We reported that calycosin shows a protective role in preventing dopaminergic (DA) neuronal cell death in PQ-exposed Canton S flies. Calycosin-fed PQ-exposed flies exhibit significant resistance against PQ-induced mortality and locomotor deficits in terms of reduced oxidative stress, loss of DA neurons, the depletion of dopamine content, and phosphorylated JNK-caspase-3 levels. Additionally, mechanistic studies show that calycosin administration improves PQ-induced mitochondrial dysfunction and stimulates mitophagy and general autophagy with reduced pS6K and p4EBP1 levels, suggestive of a maintained energy balance between anabolic and catabolic processes, resulting in the inhibition of neuronal cell death. Collectively, this study substantiates the protective effect of calycosin against PQ-induced neurodegeneration by improving DA neurons’ survival and reducing apoptosis, likely via autophagy induction, and it is implicated as a novel therapeutic application against toxin-induced PD pathogenesis.

## 1. Introduction

Neurodegenerative diseases (NDs), including Parkinson’s disease (PD), have arisen as one of the greatest clinical challenges of the 21st century [1,2]. PD has been reported as the second most common age-related movement disorder, affecting ~3.9% of the world’s population over 60–65 years of age [3]. It is primarily characterized by progressive dopaminergic (DA) neuronal loss in the substantia nigra pars compacta region of the midbrain, the aggregation of α-synuclein, and the presence of Lewy bodies, which are the defining pathological characteristics of the disease [4]. As a global disease burden, NDs cost the U.S. economy billions of dollars each year in direct health care treatment costs, and the cost to society will further and substantially increase. Unfortunately, the intricate pathogenesis of idiopathic PD is still not well understood, and the current therapies, involving the administration of dopamine precursors, dopamine agonists, anticholinergic agents and levodopa (L-DOPA), provide only symptomatic relief for a few years, and fail to prevent or slow down the disease’s progression [5,6], because their long-term use is frequently linked with a progressive decrease in the drugs’ effect, motor fluctuations, dyskinesias, and drug-associated toxic insult [5]. Thus, the intricate PD pathology and the paucity of long-term treatment possibilities continue to be major limitations in PD treatments. This scenario has attracted increasing attention from researchers to investigate novel treatment approaches targeting the early molecular signaling that is involved in PD development and progression.

In addition to the PD cases with genetic mutations, earlier epidemiological reports have strongly demonstrated that occupational exposure to environmental toxins, including pesticides such as paraquat (PQ), rotenone, MPTP, or other neurotoxic-specific chemicals, is associated with an increased risk of developing PD [7,8]. Exposure to PQ is reported to induce oxidative stress (OS) and mimic pathological symptoms similar to PD, including impaired locomotor function and selective DNergic neuronal loss in both *Drosophila* and mammalian models [9,10,11,12]. Recently, Stykel et al. [13] demonstrated the promising link between PQ exposure and PD development by using human pluripotent stem cells. Numerous studies have reported that OS, mitochondrial dysfunction, autophagy disruption, and cell death are major mechanisms associated with the PQ-induced neurotoxicity in exposed organisms [14,15,16,17]. Mitochondrial dysfunction is the key source of ROS generation, and also a key target organelle for OS [18,19]. PQ-induced mitochondrial dysfunction was characterized by lower mitochondrial membrane potential (MMP) and ATP production, resulting from excessive OS and mitophagy impairment with an abnormal presence of autophagy intermediaries (autophagosomes or autolysosomes) or inhibition.

Autophagy is a lysosome-driven process to get rid of damaged cellular organelles, protein aggregates, and toxic cargos that lead to cellular dysfunction. Alterations in endocytic pathways may obstruct the turnover of the autophagosome complex and lead to diseases. In the last decade, the basic function of autophagy in neuronal homeostasis has been reported, as its dysfunction or dysregulation caused neurodegeneration [20,21]. Reportedly, the impaired clearance of autophagic vacuoles can result in locomotor and behavioral alterations, as well as the inclusion of polyubiquitinated proteins aggregates in neurons, and huge neuron loss was observed in the CNS of animal models and also in post-mortem brain samples of PD patients [19,20,21,22,23]. In addition, defective autophagy increased PQ-mediated neurotoxicity, whereas its activation attenuated this adverse effect [14,22,23,24,25].

Several studies have reported the use of plant-derived molecules as templates for the development of novel medicines for many clinical diseases, including NDs. L-dopa, currently prescribed as the most commonly used medicine for PD treatment, was first extracted from the *Vicia faba* bean. In addition, other natural molecules produced from plants and microbes have been associated with decreased mortality and protective effects against toxin-induced neurodegeneration—for instance, fisetin, rapamycin, metformin, catalpol, gardenine-A, vasicinone, curcumin and its derivatives, quercetin and 7,8-dihydroxyflavone [24,26,27,28,29,30,31,32]. However, the neuroprotective molecular mechanisms of the flavonoid compounds are yet not fully clear. In addition, most of the studies focused on assessing the neuroprotective efficacy in cell culture models using super-physiological concentrations of phytonutrients, which mostly produce poor clinical results. There is a necessity to examine the neuroprotective efficacy of phytochemicals at their pharmacologically relevant concentrations in the context of living organisms.

In the present study, we employed *Drosophila melanogaster*, which is a widely accepted PQ-induced PD model [11,12,28,33], as an in vivo screening platform to investigate the neuroprotective efficacy of natural compounds on PD. Plants produce several compounds, many of which interact with evolutionarily conserved signaling pathways, as these molecules evolved either with a common ancestor or in response to insect herbivory. Moreover, the *Drosophila* model of PD gave insights into molecular pathways affected in the early phase of PD development, thus showing the *Drosophila* PD model to be a resourceful and economical screening platform during the initial phases of the drug discovery pipeline before validation in mammalian models. Here, we investigated the neuroprotective efficacy of calycosin, an isoflavonoid phytoestrogen extracted from *Astragalus membranaceus*, against PQ-induced neurodegeneration, which is not merely based on anti-inflammatory activities. Earlier studies reported that calycosin exhibited neuroprotective functions on cerebral ischemia and reperfusion-induced neurological injury, intracerebral hemorrhage and high glucose-induced oxidative stress and neuroinflammation, as well as neuronal apoptosis [34,35,36,37]. Yang et al. [38] also reported that calycosin administration mitigates MPTP-induced PD-like conditions in mice by inhibiting the activation of TLR-NF-κβ and MAPK signaling-mediated inflammatory responses. Moreover, Pan et al. [39] also showed that calycosin exerted its neuroprotective functions against the increased accumulation and formation of α-synuclein amyloid-induced neurotoxicity through its antioxidant properties. However, other mechanisms through which calycosin can exert anti-neurodegenerative functions are currently unclear.

Hence, in this study, calycosin is shown to confer protection against PQ-induced neurodegeneration, as revealed by lower neurodegeneration, better locomotor performance, and increased fly survival, primarily governed by modulating OS signaling and neuronal cell death, improving mitochondrial functions, and restored autophagy in exposed organisms.

## 2. Materials and Methods

### 2.1. Fly Strains and Culture

All fly strains used in this study were cultured at 24 ± 2 °C with 12 h alternating light–dark cycles on *Drosophila* standard food medium with agar-agar (10.46 gm), glucose (27.88 gm), sugar (28.97 gm), maize (47.89 gm), yeast (63.46 gm), propionic acid (10.46 mL), and methyl-paraben (13.11 mL) as constituents for 1 L of food. *Canton S* was used as the wild-type strain (Bloomington stock (BS) number #64349), hereafter referred to as wild type. *UAS-mitoQC* (BS no. #91641) and *TH-Gal4* (BS no. #8848) (a dopaminergic neuron specific Gal4 driver) lines were obtained from the Bloomington stock center, Bloomington, IN, USA.

### 2.2. Determination of Calycosin Concentration

To determine the calycosin concentration, 3–5-day-old male flies were exposed daily to 5 mM paraquat (PQ) and different concentrations of calycosin (0.0, 100.0, 200.0, and 400.0 µM) in filter paper with 5% sucrose only. Based on the survival assay and locomotor activity of surviving flies (please see details in Section 2.4), we evaluated 100.0 µM as the optimal concentration and 48 h exposure to calycosin throughout the study.

### 2.3. PQ Exposure and Calycosin Treatment Schedule

Three-to-five-day-old *Canton S* male flies were divided into the following groups: (1) control; (2) control plus calycosin (100.0 µM); (3) 5 mM PQ and (4) PQ plus calycosin (100.0 µM). For PQ and calycosin exposure, we used 5% sucrose and 1.3% agar-agar as the treatment medium. Three to five-day-old male flies were starved for 6 h in only agar containing vials, and after that, all flies were transferred into agar, sucrose and 5 mM PQ with or without calycosin (100.0 µM) for 48 h. Control flies were transferred into those vials that contained only sucrose and agar. A 5-mM PQ concentration was selected for the study of neurotoxic effects in *Drosophila,* following earlier studies [28,33,40].

### 2.4. In Vivo Assays

#### 2.4.1. Survival Assay

Survival assays were performed by scoring the number of living flies daily until the end of the exposure period (5 days). In brief, 20 3–5-day-old male flies, after 6 h of fasting, were collected in 5 vials containing filter paper soaked with 180.0 µL solutions consisting of 5% sucrose and 5 mM paraquat (PQ) with or without different concentrations of calycosin (0.0, 100.0, 200.0 and 400.0 µM), while control flies received only 5% sucrose. Solutions were transferred every alternate day until all the flies were dead. The numbering of all living flies was performed after every 24 h, and each set of experiments was carried out in triplicate.

#### 2.4.2. Climbing Assay

We assessed the locomotor performance of unexposed and PQ-exposed fly groups fed with or without calycosin (100 µM) using the negative geotaxis behavior assay, as reported earlier [11]. In brief, 20 male flies were randomly selected from each exposure group and placed in a vertical plastic tube (25 cm length × 2 cm width). After 1 min of acclimatization to the environment, flies were gently taped to the bottom of tube, and we scored those flies climbing over a 10 cm distance (n_top_) and those that remained below this mark within 10 s separately. Five trials were carried out in each 1-min time interval, and three independent biological replicates were used for each fly group. Climbing activity was calculated using the formula (1/2[(n_tot_ + n_top_ − n_bot_)/n_tot_), presented as the locomotor performance index (PI).

### 2.5. Ex Vivo Assays

#### 2.5.1. Biochemical Assays of Oxidative Stress Parameters

At the end of exposure, live flies (~30/group) were immobilized by being kept on ice for a short period, and their heads were separated from their bodies using needles. Thereafter, the heads were homogenized in pre-chilled 0.1 M phosphate buffer (pH 7.4) supplemented with 0.15 M KCl to the constituent 10% homogenate. Following this, centrifugation was performed at 12,000× *g* for 10 min at 4 °C, the pellet was discarded, and the obtained supernatant was used for different assays.

#### 2.5.2. Assessment of General ROS Level

The general level of ROS was determined in 10.0% tissue homogenate using a dye, 2′,7′-dichlorodihydrofluorescein diacetate (Catalog number: C6827, Invitrogen, Waltham, MA, USA), which was latter oxidized by ROS into highly fluorescent 2′,7′-dichlorofluorescein (DCF) according to the method of Zhao et al. [41] with minor modifications. In brief, 100.0 μL of the homogenized sample was incubated with 10.0 μM DCFH-DA in the dark at room temperature for 1 h. The increased relative DCF fluorescence intensity was measured with excitation and emission wavelengths of 485 and 535 nm, respectively, by using the scanning unit of the Flx800 fluorescence microplate reader (Bio-Tek Instruments, Wokingham, UK).

#### 2.5.3. Measurement of Intracellular Superoxide (O_2_^•−^) Radical Level (Quantitative)

For the detection of O_2_^•−^ level in head tissue homogenate by staining with dihydroethidium dye (DHE; Catalog number: D11347, Invitrogen, Waltham, MA, USA), a procedure similar to that of Zhao et al. [41] was followed with minor variations. In brief, DHE with a 10 µM final concentration was added to the tissue homogenate and then incubated in the dark for 30 min at 25 ± 2 °C. The relative fluorescence intensity of DHE oxidation (presented as fold change) was measured at 435-nm excitation/530-nm emission wavelengths by using a Flx800 fluorescence microplate reader (Bio-Tek Instruments, Wokingham, UK).

#### 2.5.4. Assay for In Vivo Detection of O_2_^•−^ Radicals in Fly Brain Tissue

For the measurement of in vivo O_2_^•−^ radical formation by means of staining with DHE dye, Owusu-Ansah, et al. [42] protocol was followed. In brief, brain tissues (10 nos.) from unexposed and exposed fly groups were dissected in Schneider’s insect medium (Catalog number: S0146, Sigma-Aldrich, Millipore, St. Louis, MO, USA) at room temperature. After that, tissues were placed in the 1XPBS containing 30.0 μM DHE for 5 min at 25 ± 2 °C under dark condition. After staining, samples were washed three times briefly with 1XPBS, and then directly transferred onto a glass slide in a drop of 1XPBS for visualization, and their analysis was performed using a confocal laser-scanning microscope (Leica TCS-SPE, Nussloch, Germany). The intensity of DHE staining was quantified using ImageJ software.

#### 2.5.5. Measurement of Peroxynitrite (ONOO^−^) Radical Level

To measure the ONOO^−^ radical level in head tissue homogenate, dihydrorhodamine 123 (DHR; Catalog number: D632, Invitrogen, Waltham, MA, USA) was used. In brief, 50 µL of tissue homogenate was incubated with 450 µL of 1XPBS containing 20 µM of DHR 123 for 10 min at 25 ± 2 °C under dark conditions. After that, the mean fluorescence intensity of DHR 123 (presented as fold change) was determined at 500-nm excitation/536-nm emission wavelengths using a Flx800 fluorescence microplate reader (Bio-Tek Instruments, Wokingham, UK).

#### 2.5.6. Assays for Enzymatic and Non-Enzymatic Oxidative Stress Parameters

##### Superoxide Dismutase (SOD) Activity Assay

SOD activity was examined according to the earlier published method of Nishikimi et al. [43] with slight modifications, as presented by Gupta et al. [44]. In brief, the total sample mixture (3.0 mL) was prepared by adding double distilled water (DDW), sodium pyrophosphate buffer of pH 8.3, nitroblue tetrazolium (NBT), phenazine methosulphate (PMS), reduced nicotinamide adenine dinucleotide (NADPH) and 10% brain tissue samples homogenate. The enzyme concentration (50%) that inhibits chromogen production in 1 min was defined as one unit (absorbance at 560 nm), and the data were expressed as units min^−1^ mg fly head protein^−1^.

##### Measurement of Catalase (CAT) Activity

We estimated CAT activity via measuring the rate of hydrogen peroxide (H_2_O_2_) dissociation in the brain tissue sample following the earlier published method of Sinha et al. [45]. Briefly, the sample mixture contained DDW, 0.01 M phosphate buffer (pH 7.0) and 10.0% tissue homogenate. Then, we added dichromate (5% K_2_Cr_2_O_7_) and acetic acid (1:3 volume acetic acid solution) and H_2_O_2_ (0.2 M) reagents to start and stop the reaction, respectively. The rate of H_2_O_2_ concentration reduction was quantified at 570 nm by using DDW as a blank. CAT activity (U) was presented as µmoles H_2_O_2_ min^−1^ mg fly head protein^−1^.

##### Lipid Peroxidation (LPO) Assay

Lipid peroxidation (LPO) was assayed in terms of MDA content as an end product by measuring 1,1,3,3 tetraethoxypropane as an external standard according to Ohkawa et al.’s [46] procedure, and the levels of LPO were presented in terms of nmoles MDA formed h^−1^ mg^−1^ fly head protein.

##### Measurement of Protein Carbonyl (PC) Content

We measured the PC content in head homogenate samples from control and treated groups according to the procedure as reported by Mesquita et al. [47]. In brief, 50 µL of tissue homogenate was incubated with 50 µL of di-nitro phenyl hydrazine (DNPH) (10 mM dissolved in 2 M HCl) in a 96-well plate for 10 min at 25 ± 2 °C under dark conditions. Then, 25 µL of 6 M NaOH was added, and the homogenate was re-incubated again at 25 ± 2 °C for 10 min. After that, the relative PC content (expressed as arbitrary units) was measured at 370 nm using 2 M HCL as a blank, and the data were normalized to values obtained for the control group.

##### Estimation of Reduced Glutathione (GSH)

We measured reduced glutathione (GSH) content in the brain tissue by using Elman’s reagent [48]. The reaction mixture had 5.0% TCA, 0.2 M of sodium phosphate buffer (pH 8.0), 0.01% 5, 5′-dithiobis-2-nitro benzoic acid (DTNB), and tissue homogenate. The absorbance was measured at 412 nm, and the proportion of GSH content was expressed as nmoles mg protein^−1^.

### 2.6. Immunohistochemistry

Whole brains were dissected, and the counting of their dopaminergic neurons was performed as previously reported [11]. In brief, whole brain tissues from unexposed and exposed fly groups were dissected in 1XPBS after pre-fixation with 8% paraformaldehyde (PFA) for 10 min at room temperature. Next, brain samples were fixed in 4% PFA for 30 min and then washed three times with 1XPBS-T (0.1% Triton X-100) each for 10 min. After blocking with 5% heat-inactivated fetal bovine serum (FBS) in 1XPBS-T solution for 2 h at 25 ± 2 °C, the tissues were then incubated in primary antibody anti-tyrosine hydroxylase (1:100) (Catalog number: AB152, Sigma-Aldrich, Millipore, St. Louis, MO, USA) for two days at 4 °C. After being washed three times in 1XPBST, tissues were incubated with Alexafluor-488 goat anti-rabbit (1:200; Invitrogen, Carlsbad, CA, USA) secondary antibodies in 5.0% FBS for 2 h at 24 ± 1 °C. Finally, tissue samples were mounted with Vectashield mounting media (Vector Laboratories, Germany) for 10 min at room temperature and images were captured on a confocal laser scanning fluorescence microscope (Leica TCS-SPE Microsystems, Wetzlar, Germany) using a 20X objective. For the counting of DTH-positive neurons within each major DN cluster, individual brain hemispheres were observed by using ImageJ cell counter plugin software (National Institute of Health, Bethesda, MD, USA). Finally, the images were processed using Adobe Photoshop 8.0 software.

### 2.7. Dopamine Measurements

After the 48 h of exposure, 50 flies/samples, *n* = 3, were frozen at −80 °C, and their heads were separated from their bodies using sharp needles. Thereafter, the heads were homogenized in pre-chilled 0.1 M perchloric acid (pH 7.4), and, following this, centrifugation was performed at 12,000× *g* for 15 min at 4 °C. The pellet was discarded, and the obtained supernatant was used immediately or stored at −80 °C for 24 h until use. A dopamine ELISA kit (Catalog number: EU0392, Fine Test^®^, Wuhan, China) was used to estimate DA levels in the supernatant samples according to the manufacturer’s procedure. The Flx800 fluorescence microplate reader (Bio-Tek Instruments, Wokingham, UK) was used to measure the absorbance at the 450-nm wavelength, and the proportion of DA content was presented as ng/mg protein.

### 2.8. Tyrosine-Hydroxylase (TH) Enzyme Kinetic Assay

For the measurement of TH enzyme activity, Vermeer et al.’s [49] method was followed with slight modifications. This enzyme assay depends on the production of chromopore dopachrome via the reaction occurring between L-DOPA as formed by TH and sodium periodate (NaIO4). In brief, NaIO4 (200 μM) was first prepared in distilled water, where it served as the substrate to react with L-DOPA, and then 100 µL aliquots were added into a 96 well plate and kept at room temperature for 10 min. Next, 20 µL samples of head homogenates were mixed with 80 µL of reaction buffer (100 mM HEPES (pH 6.8), 200 μM L-tyrosine, 500 μM FeSO4, and 0.25 mM BH4) to form the reaction mixture. Thereafter, 100 µL of the reaction mixture was added to a 96-well plate with NaIO4, and immediately TH activity was measured at 475-nm absorbance every 30 sec for 10 min at 37 °C using the Flx800 fluorescence microplate reader (Bio-Tek Instruments, Wokingham, UK). The formation of L-DOPA was expressed in terms of nmoles/min/mg protein depending on the molar absorbance coefficient for dopachrome ε = 3700 M^−1^ cm^−1^.

### 2.9. Western Blotting

For the analysis of protein expression, the procedure of Chaouhan et al. [50] was followed with minor modifications. Briefly, the heads of 30 male flies were homogenized in protein extraction buffer ((2% SDS, 150 mM NaCl, 50 mM Tris, pH 7.5)) supplemented with a cocktail of proteinase-K inhibitor and phosphatase inhibitor, and then were kept for 30 min on ice for complete protein extraction. After centrifugation was performed at 14,000× *g* for 10 min at 4 °C, the obtained supernatant was transferred to a new Eppendorf tube and the pellet was discarded. The supernatant was further used for the protein estimation (please see the protocol in Section 2.11) and the Western blotting assay mentioned below or was stored at −20 °C till further use. An equal amount of protein (~40.0 µg) was boiled (95 °C, 5 min) with an equal amount of 2X sample buffer (100 mM Tris-Cl (pH 6.8); 4% SDS; 0.02% bromophenol blue; 20% glycerol; and 200 mM β-mercaptoethanol) and then was separated by using of 12–15% SDS-polyacrylamide gel electrophoresis (SDS-PAGE) at a constant 80 volts for an appropriate time. After the electrophoresis, polypeptides were transferred onto polyvinylidene difluoride (PVDF) membrane (Millipore Corporation limited, Bedford, MA, USA) in a transfer buffer (25 mM Tris base, 190 mM glycine, and 20% methanol) using a Bio-Rad wet dry transfer system (Bio-Rad Laboratories, Bedford, MA, USA), followed by incubation in a blocking buffer solution (5% *w/v* nonfat dry skim milk powder, 1 × transfer buffer saline (TBS), and 0.1% Tween-20) for 2 h at room temperature. After the blocking and hybridization steps, the membrane was incubated with indicated primary antibodies with appropriate dilution (for detailed information see in Table 1) at 4 °C for overnight. Subsequently, HRP-conjugated anti-rabbit and anti-mouse secondary antibodies (1:3000) were applied, followed by the detection of chemiluminscence signals using Super Signal™ West Femto Maximum Sensitivity Substrate detection reagent (Catalog number: 34095; Thermo Fisher Scientific, Waltham, MA, USA) on an ImageQuant LAS4000 Imager system (GE Healthcare, Chicago, IL, USA). Finally, quantification of protein blots was carried out with the ImageJ program, with *β*-tubulin (1:1500) used as a loading as well as an endogenous control.

### 2.10. Analysis of Protein Oxidation by an Immunoblotting Method

We measured oxidative modification in cellular proteins in the fly brain tissues of chemically exposed groups using the OxyBlot protein oxidation detection kit (Catalog number: ab178,020 abcam, Cambridge, MA, USA) as per the manufacturer’s instructions with minor changes. In brief, dissected brain tissues (10 nos.) were homogenized in 10.0 µL of lysis buffer (20.0 mM Tris (pH 6.8), 6.0% SDS, 2.0% β-mercaptoethanol) and solubilized with an equal volume of 2X extraction buffer. Following incubation at 25 ± 1 °C for 20 min, the solubilized homogenate was then derivatized with 20.0 µL of 1X DNPH solution. After 20 min incubation, the reaction was stopped by means of the addition of 10.0 µL neutralization solution. Protein samples (20.0 µL of total fraction) were electrophoresed using 12% SDS-PAGE. After electroblotting, the membrane was then probed with an anti-DNP antibody (anti-rabbit, 1:5000; Catalog number: ab178020 abcam, Cambridge, MA, USA) followed by incubation with an HRP-conjugated goat anti-rabbit 2° antibody (1:5000, abcam, Cambridge, MA, USA). Finally, the visualization and analysis of immunoblots were performed as mentioned above.

### 2.11. Protein Measurement

The protein concentration in the head homogenate was measured by using the Bradford reagent (1:5 dilution) (Bio-Rad laboratories, Hercules, CA, USA) and was calculated using bovine serum albumin (BSA) as a standard (2.0 mg/mL) prepared under similar experimental conditions. In brief, the BSA standard protein curve was prepared with a range of different protein concentrations (0.0, 0.1, 0.2, 0.3, 0.4, and 0.5 mg/mL) from the BSA stock concentration (2.0 mg/mL). Further, 2.0 µL protein samples were mixed with 198.0 µL of Bradford reagent in a 96-well plate. After 10 min of incubation at room temperature, the optical density (OD) of the protein samples was measured at an absorbance of 595 nm using a multiwall ELISA plate reader (Bio-Tek Instruments, Wokingham, UK), and protein concentrations were calculated.

### 2.12. Assessment of Apoptosis

#### 2.12.1. Biochemical Methods (Assays of DEVD- and IETD-Ase Activity)

To determine caspase-3 (*DEVDase*) and caspase-9 (*IETDase*) activity in a fly head homogenate from unexposed and PQ-exposed groups fed with or without calycosin (100 µM), we used a colorimetric assay kit (Bio Vision Inc., Milpitas, CA, USA) as per the manufacturer’s protocols. In brief, 50 µL of the collected supernatant from the tissue homogenate was incubated at 37 °C for 2 h with 50 µL of chilled lysis buffer, 2X reaction buffer containing 10.0 mM dithiothreitol, and 200 µM caspase-3 substrate (Ac-DEVD-*pNA*) and caspase-9 substrate (Ac-LEHD-*pNA*). The reaction rate of the yellow color being formed was measured 405-nm absorbance against a blank by using a Flx800 fluorescence microplate reader (Bio-Tek Instruments, Wokingham, UK). Finally, the fold increase in caspase-3 and -9 activity was calculated and directly compared to the level of the control.

#### 2.12.2. In Vivo Imaging

For the examination of caspase-3 expression in neuronal cells of unexposed and PQ-exposed groups fed with or without calycosin (100 µM), dissected brain tissues (~10 nos.) were fixed as described above (please see Section 2.6) and stained with cleaved caspase-3 primary antibody (1:100) and an appropriate secondary antibody (1:400). After that, samples were stained with Alexa Fluor 488^®^ phalloidin (1:200; Catalog number: A12379; Invitrogen) for 5 min in the dark at 25 ± 2 °C, and then were mounted with Vectashield mounting media (Vector Laboratories, Germany) for 10 min at room temperature. Finally, images were processed using a confocal laser scanning fluorescence microscope (Leica TCS-SPE Microsystems, Wetzlar, Germany) with 63X objective, focusing on a specific lobe of each brain.

### 2.13. Assessment of Mitochondrial Function

All the mitochondrial functions were determined in the fly head homogenate of unexposed and PQ-exposed groups fed with or without calycosin (100 µM) except for those in vivo mitochondrial membrane potential and mitochondrial O_2_^•−^ radical assays. For mitochondrial membrane potential and mitochondrial O_2_^•−^ radical assays, we used whole brain tissues followed the published standard procedures.

#### 2.13.1. ATP Measurement

The ATP content was determined in homogenized head samples from unexposed and PQ-exposed fly groups fed with or without calycosin (100 µM) according to the published method of Tennessen et al. [51]. In brief, for ATP content estimation, ~30 heads were homogenized in 100 µL of ATP extraction buffer (6 M guanidine-HCL with 100 mM Tris and 4 mM EDTA, pH 7.8) to assess the inhibition of ATPase. Following centrifugation at 8000× *g* for 10 min at 4 °C, 10.0 µL of the collected supernatant was used for protein estimation, while the remaining part was kept for the ATP assay. Before homogenization, the ATP assay reaction mix (Catalogue no.: A22066 Molecular Probes ATP kit, Eugene, OR, USA) was prepared by mixing 3.56 mL ddH_2_O, 200.0 µL of 20X reaction buffer, 40.0 µL of 0.1 M DTT, 200.0 µL of 10 mM D-luciferin, and 1.0 µL of firefly luciferase. Furthermore, the ATP assay reaction was initiated by adding 90.0 µL of the ATP reaction mix and 10.0 µL of the homogenized sample, and then, immediately, luminescence was measured by using an Flx800 fluorescence microplate reader (Bio-Tek Instruments, Wokingham, UK). The ATP standard curve was assessed to determine its level in the head of each sample and the data were normalized by estimating the protein concentration in each sample.

#### 2.13.2. Measurements of Mitochondrial Membrane Potential (MMP, Δψm)

##### Biochemical Method

We employed the membrane-permeant JC-1 dye (5,5′,6,6′-tetrachloro-1,1′,3,3′-tetraethyl–benzimidazolyl-carbocyanine iodide) (Catalogue no: M34152; Invitrogen, Carlsbad, CA, USA) to measure membrane potential in homogenized head samples from unexposed and PQ-exposed groups fed with or without calycosin (100 µM) according to the published method of Wang et al. [52]. Briefly, 30 heads were homogenized in chilled isolation buffer (5 mM Tris-HCl, 2 mM EGTA and 250 mM sucrose, pH 7.4), and differential centrifugation followed (200× *g* for 5 min and 10,000× *g* for 10 min) at 4 °C. Next, isolated mitochondria were resuspended in Tris-EDTA buffer (50 mM Tris-Cl + 0.1 mM EDTA (pH 7.4)). To measure MMP in homogenized samples, isolated mitochondrial fractions were incubated with 2 µM JC-1 dye for 10 min at room temperature. Finally, absorbance was taken first at 410 and 529 nm excitation and emission wavelengths, respectively, and after that, at 410/590-nm excitation and emission wavelengths, respectively. The MMP fluorescence intensity was calculated by taking the ratio of emission wavelengths at 590 nm to the 529 nm.

##### In Vivo JC-1 MMP Staining

JC-1 MMP staining was performed according to a previously published protocol [53]. In brief, whole brains (~10 nos.) were dissected in 1XPBS, and thereafter stained with JC-1 dye (10.0 µM final concentration) (Catalogue no.: T3168; Invitrogen, Carlsbad, CA, USA) for 30 min at room temperature. After washing three times with 1XPBS for 5 min, samples were mounted in Vectashield mounting media (Vector Laboratories, Germany) and, immediately, samples were imaged using a confocal laser scanning fluorescence microscope (Leica TCS-SPE Microsystems, Wetzlar, Germany) with 20X objective. The ratio of red and green fluorescence intensity from JC-1 dye was calculated by ImageJ software.

#### 2.13.3. Mitochondrial Complex I and III Enzyme Activity Assay

The respiratory complex enzyme activity assays were carried out according to previously published procedures [54]. In brief, for the analysis of the mitochondrial complex I assay, 1 µg of the mitochondrial fraction was added to 100.0 µL of NADH dehydrogenase reaction buffer (50 mM PBS (pH-7.4), 2.5 mg/mL BSA, 70 µM decyloubiquinone (dUB), 250 µM KCN, 25 µM antimycin A and 2 µM rotenone) in a 96-well plate, and then kept for 10 min at room temperature. After that, the assay reaction was initiated by the addition of 200 µM NADH, and a decrease in the absorbance was measured at 340 nm.

For the analysis of complex III activity, 0.5 µg of mitochondrial proteins was added with 100.0 µL of cytochrome-C reductase reaction buffer (50 mM PBS (pH-7.4), 1.0 mg/mL BSA, 200 µM decyloubiquinone (dUB), 2 mM KCN, 250 µM EDTA and 2 µM rotenone) in a 96-well plate, and then was kept for 10 min at room temperature. After that, the assay reaction was initiated by the addition of 50 µM of oxidized *cytochrome C*, and a reduction of *cytochrome C* was measured at 550 nm.

#### 2.13.4. Measurement of Mitochondrial O_2_^•−^ Radical Generation

Mito-SOX red fluorescent dye was employed to examine in vivo mitochondrial O_2_^•−^ radical formation in whole brain tissues. Mito-SOX fluorescent dye is a live cell-permeable dye that specifically targets mitochondria and shows red fluorescence after being oxidized via O_2_^•−^ radicals. In brief, whole brains (~10 nos.) were dissected in 1XPBS and thereafter stained with Mito-SOX dye (5.0 µM) (Catalogue no.: M36008; Invitrogen, Carlsbad, CA, USA) and Mitotracker dye (100.0 nM) (Catalogue no.: M7514; Invitrogen, Carlsbad, CA, USA) for 15 min at room temperature. After washing three times with 1XPBS for 5 min, tissue samples were mounted in Vectashield mounting media with DAPI (Vector Laboratories, Germany), and immediately images of samples were captured by using a confocal laser scanning fluorescence microscope (Leica TCS-SPE Microsystems, Wetzlar, Germany) with 63X objective.

#### 2.13.5. Mitophagy Assay

For the assessment of mitophagy by mito-QC, whole brains were dissected from unexposed and PQ-exposed groups fed with or without calycosin (100 µM) of *TH-Gal4* > *UAS-mitoQC* group in 1XPBS, and they were fixed in 4% PFA for 30 min. After washing three times with 1XPBS, tissue samples were mounted with Vectashield mounting media (Vector Laboratories, Germany) for 10 min at room temperature. Finally, images were captured via sequential excitation (458 nm green; 561 nm red) and emission wavelength (578 to 678 nm) using a confocal fluorescence microscope (Leica TCS-SPE Microsystems, Wetzlar, Germany) with 63X objective.

### 2.14. Autophagosomal Study

Whole brains were dissected from unexposed and PQ-exposed groups fed with or without calycosin (100 µM) fixed as described above (please see Section 2.6), and immunostained with Atg8a primary antibody (1:100) and an appropriate secondary antibody (1:400). Next, samples were stained with 0.5 µM of Lysotracker RED DND-99 (Catalog number: L7528; Invitrogen, Carlsbad, CA, USA) for 5 min in the dark at 25 ± 2 °C, and then were mounted with Vectashield mounting media (Vector Laboratories, Germany) at room temperature for 10 min. Finally, images were processed using a confocal fluorescence microscope (Leica TCS-SPE Microsystems, Wetzlar, Germany) with 63X objective, focusing on a specific lobe of each brain.

### 2.15. Statistical Analyses

All assays were carried out three times with three independent biological replicates. Data were expressed as mean ± SD. Mean values of the data were statistically calculated by Graph Pad Prism software 5.0 (Graph Pad Inc., San Diego, CA, USA). Significant differences between mean values were analyzed by one-way ANOVA followed by Tukey’s post hoc test, and significance was represented by * *p* < 0.05, ** *p* < 0.01, *** *p* < 0.001 when compared with control flies and ^$^ *p* < 0.05, ^$$^ *p* < 0.01, ^$$$^ *p* < 0.001 when compared to the PQ (5 mM)-exposed group.

## 3. Results

### 3.1. Calycosin Alleviates PQ-Mediated Mortality and Locomotor Defects in Exposed Canton S Flies

We used an environmental neurotoxin-induced *Drosophila* neurodegenerative model that recapitulates the locomotor behaviors and other pathological symptoms of human PD as a screening platform to find nutraceuticals with neuroprotective efficacy against PQ-induced neurotoxicity. Earlier studies reported that PQ (5 mM) exposure significantly decreases survival and impairs the locomotor abilities of *Drosophila* wild-type flies [28,33,40]. In the present study, we used only adult male flies due to their greater sensitivity to PQ-induced neurotoxicity at earlier stages than female flies, while the mating status of female flies showed a different feeding pattern. In earlier findings, co-treatments of 0.5 mM levodopa, a gold standard dopamine replacement drug for PD, significantly improved survival and climbing defects after PQ-exposure in *Drosophila*, therefore supporting the therapeutic efficacy observed in human PD patients.

**Figure 1:**
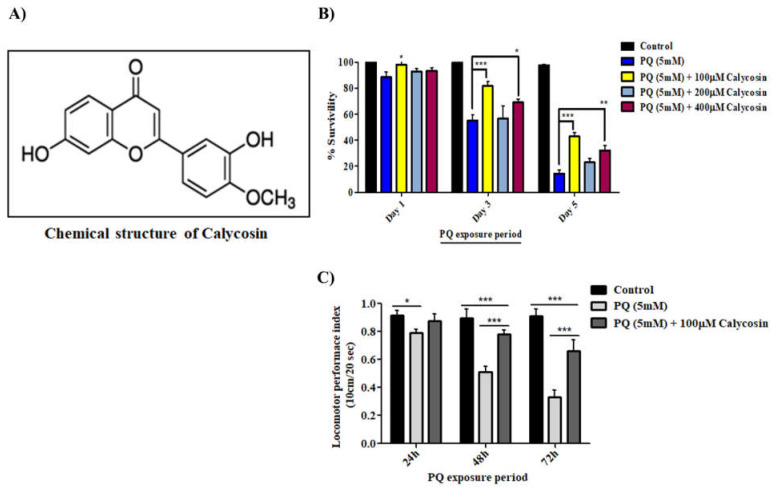
The protective effects of the phytoestrogen, calycosin against PQ-induced toxicity and locomotor defects. (**A**) The chemical structure of calycosin. (**B**) Survival assays were carried out by using 3–5-day-old adult male flies as outlined above, and the counting of surviving flies was performed every 24 h post exposure until all of the flies died, and we plotted the survival percentage graphs on days 1, 3, and 5 of PQ co-exposures with different feeding concentrations of calycosin. Data represent five independent biological replicates with 20 flies per feeding regimen. Data are presented as mean ± SD (*n* = 3); significance ascribed as * = *p* < 0.05 and *** = *p* < 0.001 vs. PQ (5 mM) exposure. (**C**) The protective effect of calycosin supplements on the flies’ locomotor performance when exposed to PQ was examined through the climbing assay. The number of flies climbing over a 10 cm distance within 10 s was recorded, and the graph was plotted at 24, 48, and 72 h. Data are presented in at least five independent experiments with 20 flies per group. Data presented are mean ± SD (*n* = 3). Significance ascribed as * = *p* < 0.05, ** = *p* < 0.01 and *** = *p* < 0.001 vs. PQ (5 mM) exposure. PQ, paraquat; SD, standard deviation.

Following a similar exposure régime, we fed 3–5-day-old adult flies with 5% sucrose solution as a control and 5 mM PQ alone or with different calycosin concentrations (0.0, 100.0, 200.0 and 400.0 µM) diluted in 5% sucrose solution on filter paper through ingestion until all flies were dead. The counting of surviving flies was performed every 24 h post-exposure, and we plotted the survival percentage graphs on days 1, 3, and 5 of PQ co-exposure with different feeding concentrations of calycosin (Figure 1). The data show that PQ with 5 mM exposure gradually significantly increase the mortality of flies, with an average of 35% and 78% on days 3 and 5, respectively. Further, we screened different concentrations and time periods of calycosin treatment and chose the 100 µM calycosin concentration for the study, as it shows maximal protection in the fly survival assays against PQ-induced toxicity. Interestingly, co-exposure of calycosin provides significant (*p* < 0.001) protection against PQ-induced toxicity, with 41% of flies surviving on day 5, compared with 22% survival being observed for PQ-exposed flies. We also performed an additional survival experiment wherein adult male flies were fed with a different concentration of calycosin (100 µM, 200 µM, and 400 µM) after 24 h, 48 h, and 72 h post PQ treatment in 5.0% sucrose solution on filter paper and check the survivality until all of the flies were dead (Figure S1). The results suggested that calycosin could also improve survival of flies pretreated with PQ and confirm that calycosin appears to be a potential protective candidate against PQ-induce neurotoxicity in flies.

Furthermore, we examined whether calycosin was capable of restoring the locomotor function impaired by PQ exposure in *Canton S* flies using the climbing assay. We fed 3–5-day-old adult male flies with 5% sucrose solution and 5 mM PQ alone or with 100 µM calycosin, and then locomotor behavior assessment was carried out at the 24-, 48-, and 72-h time points of exposure. As the data show in Figure 1C, 5 mM PQ exposure caused a significant reduction in the climbing ability of flies, with averages of 12%, 38%, and 65% at 24, 48, and 72 h, respectively. Meanwhile, calycosin exposure significantly restores locomotor abilities by 23% and 32% at 48 h and 72 h, respectively. Thus, we selected 100 µM and a 48-h exposure period to calycosin with PQ for the rest of the experiments in this study.

### 3.2. Calycosin Rescues against PQ-Induced Dopaminergic Neurons Loss in Exposed Canton S Flies

**Figure 2:**
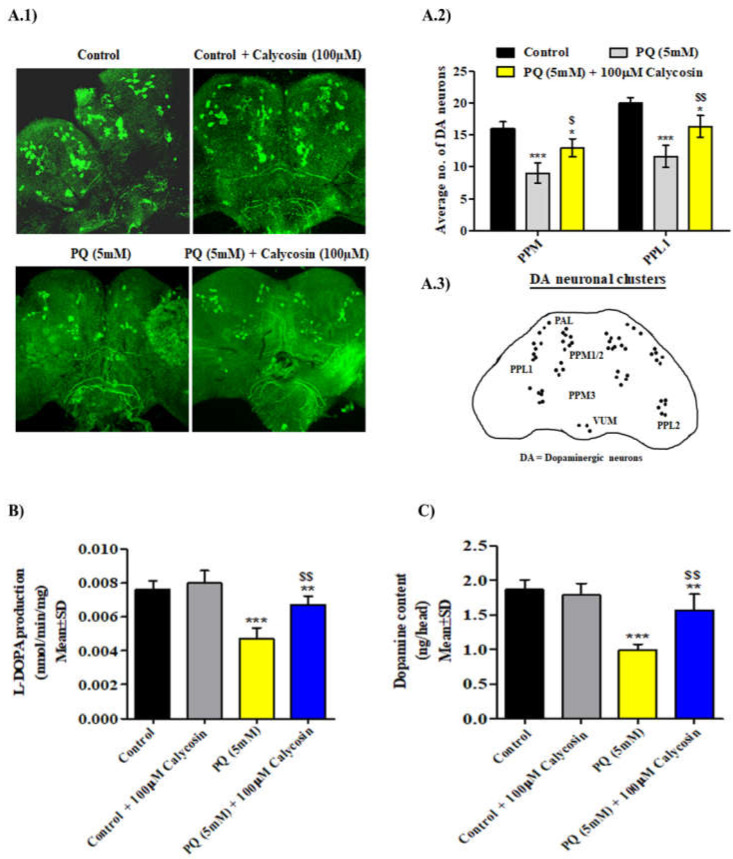
Calycosin suppresses dopaminergic neuron loss in PQ-exposed *Canton S* flies. (**A.1**) Representative confocal imaging was used to detect specific DA neuron clusters in the control flies and the flies exposed to 5 mM PQ or with PQ (5 mM) + 100 µM calycosin for 48 h. Anti-*Drosophila* tyrosine hydroxylase antibody was used to detect the specific DA neuronal clusters in the *Drosophila* brain. White and red arrows indicate the PPL and PPM DA neuron clusters, respectively. Scale bars—100 µm. (**A.2**) Graph showing the average number of DA neuron clusters in the control flies and the flies exposed to 5 mM PQ or with PQ (5 mM) + 100 µM calycosin for 48 h. (**A.3**) Schematic diagram of different DA neuron clusters. (**B**,**C**) Graph showing the Dopamine (DA) and L-DOPA production in control and 5 mM PQ or with PQ (5 mM) + 100 µM calycosin exposed flies for 48 h. Data presented are mean ± SD (*n* = 3). Significance ascribed as * = *p* < 0.05, ** = *p* < 0.01 and *** = *p* < 0.001 vs. control and ^$^ = *p* < 0.05 and ^$$^ = *p* < 0.01 vs. PQ (5 mM) exposure. DA, dopaminergic; PQ, paraquat; SD, standard deviation.

It has been reported that PQ (5 mM) exposure significantly induces dopaminergic neuron (DA) loss in both *Drosophila* and mammalian PD models [28,33,40]. Since calycosin exposure restored PQ-induced locomotor deficits, we examined the effects of calycosin on specific DA neuron clusters in the 3–5-day-old adult male flies. We analyzed DA neuron clusters using anti-tyrosine hydroxylase (TH) antibody immunostaining in DA neurons (TH, a useful marker for DA and noradrenergic neurons). Consistent with previous studies, the numbers of DA neurons in the protocerebral posterior lateral (PPL1) and protocerebral posterior median (PPM) clusters in the posterior region of the adult fly brain were observed to be significantly lower in the 48 h PQ exposure group (PPL1: 41% and PPM: 48%) than in control flies, whereas calycosin treatment resulted in a significant reduction in DA loss in the specific cluster region (PPL1: 20% and PPM: 27%) in similarly PQ-exposed flies (Figure 2A). Moreover, the observed protection of DA neurons in calycosin-fed PQ-exposed flies was evidenced via the significantly lower depletion of L-DOPA production and dopamine content as compared with that of PQ-exposed flies under a similar exposure regimen (Figure 2B,C). Therefore, these results suggest that calycosin treatment shows a potential neuroprotective role against PQ-mediated DA neuron loss.

### 3.3. Calycosin Supplements Alleviate PQ-Induced Oxidative Stress in Exposed Canton S Files

**Figure 3:**
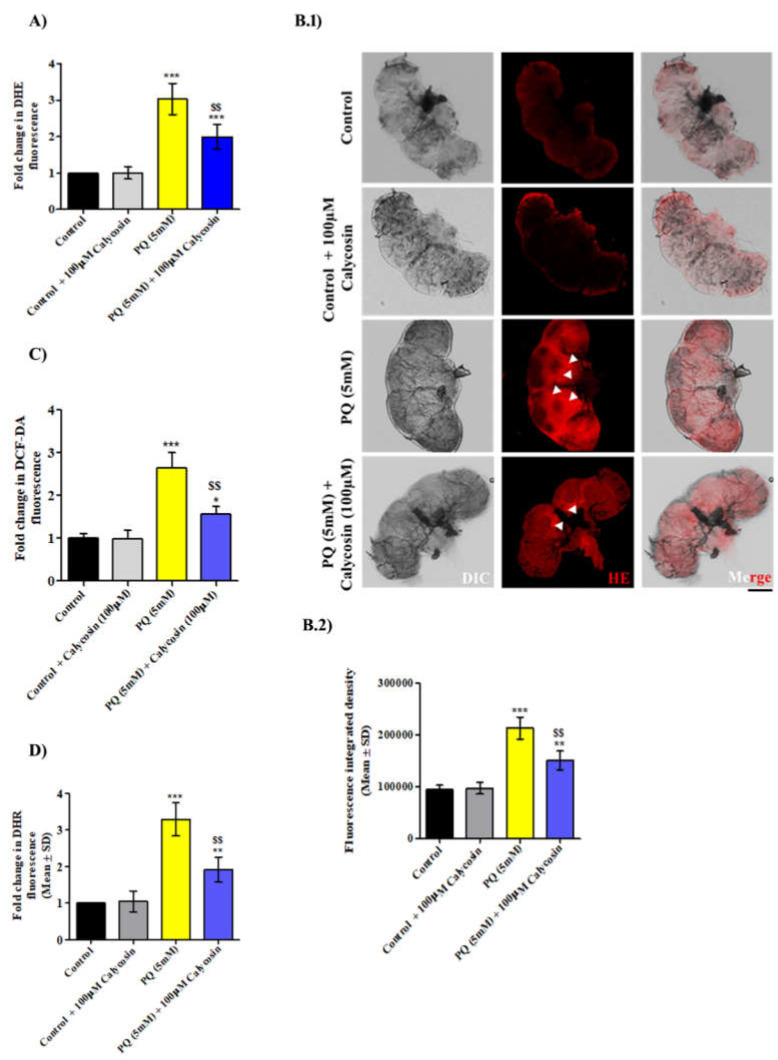
Calycosin supplements resist PQ-induced higher ROS levels. Graphical representation of ROS levels using quantitative measurement of DHE (**A**), DCF-DA (**B**) and DHR’s (**C**) fluorescence intensity in the control (**D**), 5 mM PQ and with PQ (5 mM) + 100 µM calycosin-exposed flies for 48 h. (**B.1**) Representative confocal imaging showing DHE oxidation level in live brain tissues from the control, 5 mM PQ, and with PQ (5 mM) + 100 µM calycosin-exposed flies and (**B.2**) graphs showing DHE fluorescence integrated density in control and exposed fly groups. Data presented are mean ± SD (*n* = 3). Significance ascribed as * = *p* < 0.05, ** = *p* < 0.01 and *** = *p* < 0.001 vs. control and ^$$^ = *p* < 0.01 vs. PQ (5 mM) exposure. DHE, dihydroethidium; DCF-DA, 2’,7’-dichlorodihydrofluorescein diacetate; DHR, dihydrorhodamine; PQ, paraquat; SD, standard deviation. Scale bars—100 µm.

The overproduction of ROS and oxidative stress is one of the leading causative mechanisms in PQ-induced neurotoxicity in exposed organisms. To examine whether calycosin treatment has the ability to provide protection against PQ-induced oxidative stress, the levels of ROS generation along with different oxidative stress points were examined in the brain tissues of adult *Canton S* flies after PQ exposure for 48 h. Earlier studies reported that PQ-induced neurotoxicity is specifically allied with O_2_**^−^.** generation in exposed organisms. The DHE fluorescence (a general indicator of O_2_**^−^.** formation) intensity was found to be significantly increased (~186%) in PQ-exposed flies’ brain tissue for 48 h as compared to control flies, while calycosin-fed PQ-exposed flies showed significantly reduced (~97%) DHE’s fluorescence intensity (Figure 3A,B). Furthermore, we analysed DCFDA (OH.) and DHR’s (ONOO^−^) fluorescence intensity in similarly PQ-exposed flies, wherein DCFDA (Figure 3C) and DHR’s fluorescence intensity (Figure 3D) follow a similar trend to DHE.

**Figure 4:**
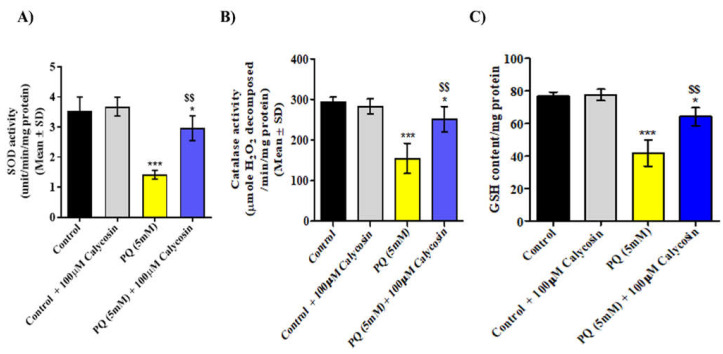

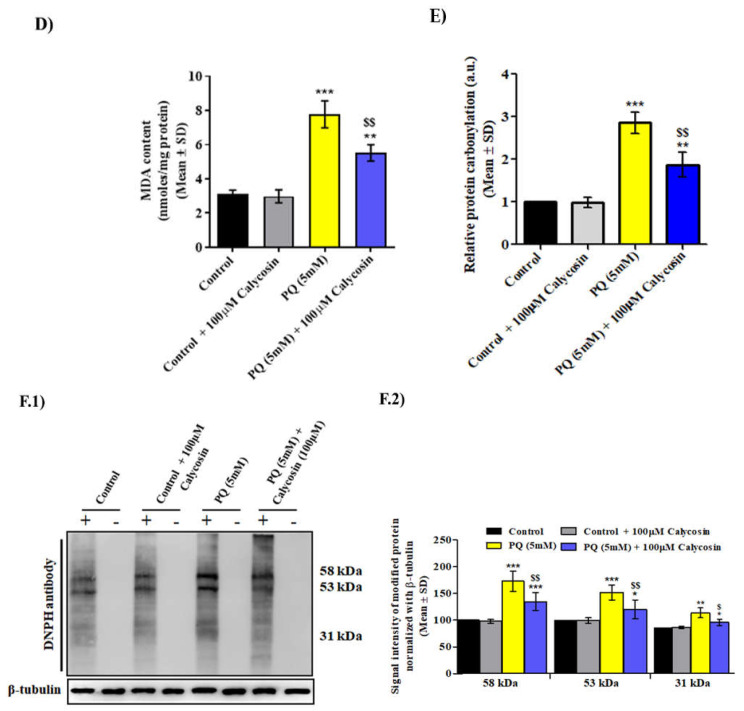
Calycosin administration optimizes the redox equilibrium in PQ-exposed flies. Graphical representation of oxidative stress (OS) end parameters such as SOD activity (**A**), CAT activity (**B**), glutathione content (**C**), MDA in terms of the lipid peroxidation (**D**) and PC content (**E**) in control, 5 mM PQ and with PQ (5 mM) + 100 µM calycosin-exposed flies for 48 h. Western blot picture (**F.1**) and its densitometric graph plot (normalized with loading control β-tubulin) showing DNP level (an indicator of oxidatively modified proteins) (**F.2**) in the protein samples from the brains of control, 5 mM PQ and with PQ (5 mM) + 100 µM calycosin-exposed flies after 48 h. The “+”- and “-” lanes represent protein samples derivatized with or without DNPH. Data presented are mean ± SD (*n* = 3). Significance ascribed as * = *p* < 0.05, ** = *p* < 0.01 and *** = *p* < 0.001 vs. control and ^$^ = *p* < 0.05 and ^$$^ = *p* < 0.01 vs. PQ (5 mM) exposure. MDA, malondialdehyde; DNPH, 2,4-dinitrophenylhydrazine; PQ, paraquat; SD, Standard deviation.

Furthermore, we monitored oxidative stress-responsive enzymes’ activity: SOD and CAT were significantly reduced in PQ-exposed flies after 48 h, and the same were significantly higher in calycosin-fed PQ-exposed flies compared with PQ alone-exposed flies (Figure 4A,B). Since O_2_**^−^.** is a substrate of SOD activity, it can be assumed that higher O_2_**^−^.** formation may lead to the induction of higher SOD activity in PQ-exposed flies. Interestingly, decreased SOD activity was observed in PQ-exposed flies, which may be due to higher ONOO^−^ formation in cells causing the nitration of SOD, which leads to the moderation of its activity. Along with the reduced ROS generation and higher SOD and CAT activity observed in calycosin-fed PQ-exposed flies, a significantly lower depletion of GSH content (~26%) and decrease in MDA (~33%) and PCC content (~29%) were found in their brain tissues as compared with similarly PQ-exposed flies (Figure 4C,D). The level of oxidatively modified proteins (using a DNP-specific antibody) in PQ-exposed flies shows a significantly higher DNP-specific signal intensity (at ~58 kDa, ~53 kDa and ~31 kDa), whereas a lower intensity was observed in calycosin-fed PQ-exposed flies as compared to similarly PQ-exposed flies. Therefore, treatments of calycosin administered to the PQ-exposed flies seems to have offered protection against neuronal cell damage due to lower levels of ROS generation and oxidative stress.

### 3.4. Calycosin Supplements Lessens PQ-Induced Caspase Dependent Neuronal Cell Death Response in Exposed Canton S Files

**Figure 5:**
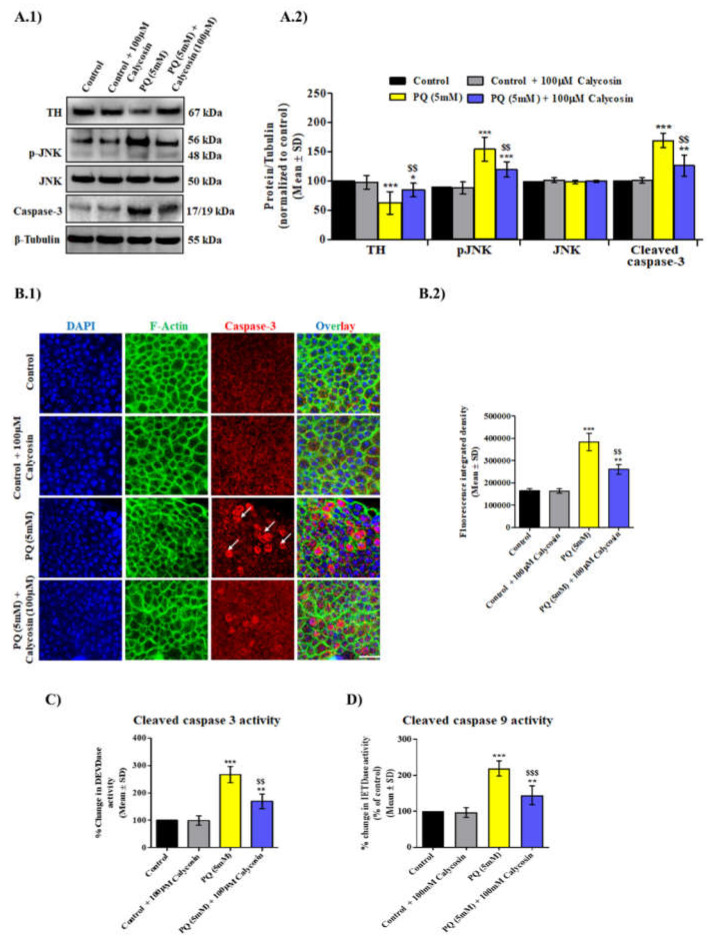
Flies with calycosin supplements were more resistant to neuronal cell death after PQ exposure. Representative immunoblot images (**A.1**) and the densitometry analysis graph (**A.2**) of TH, pJNK, JNK and cleaved capase-3 in the protein samples from the brains of control, 5 mM PQ and with PQ (5 mM) + 100 µM calycosin-exposed flies after 48 h. Values are represent mean ± SD (*n* = 3). Significance ascribed as * = *p* < 0.05, ** = *p* < 0.01 and *** = *p* < 0.001 vs. control and ^$$^ = *p* < 0.05 vs. PQ (5 mM) exposure. (**B.1**) Representative confocal images showing localization of cleaved caspase-3 in the neuronal cells using F-actin staining in brain samples of control, 5 mM PQ and with PQ (5 mM) + 100 µM calycosin-exposed flies after 48 h and (**B.2**) graphs showing fluorescence integrated density in control and exposed fly groups. Graphs showing (**C**) Cleaved caspase-3 and (**D**) cleaved caspase-9 activity in control, 5 mM PQ and with PQ (5 mM) + 100 µM calycosin-exposed flies after 48 h. Values represent mean ± SD (*n* = 3). Significance ascribed as ** = *p* < 0.01 and *** = *p* < 0.001 vs. control and ^$$^ = *p* < 0.05 and ^$$$^ = *p* < 0.001 vs. PQ (5 mM) exposure. PQ, paraquat; SD, standard deviation. Scale bars—20 µm.

Earlier studies have reported that the involvement of PQ-mediated JNK activation and elevated cleaved caspase-3 levels in inducing DA neurons’ apoptosis leads to a PD-like pathogenesis in exposed organisms, and it was observed that upon acute exposure to PQ, the flies showed increased mortality rate and OS levels [11,12,30,35]. Furthermore, the inhibition of caspases has been found to lessen the death of DA neuron cells in mammalian and invertebrate PD models. Flavonoids are well known to regulate apoptotic signaling in both in vitro and in vivo ND models [30,55]. Thus, we aimed to examine the protective effects of calycosin against PQ-induced neuronal cell death. Western blot analysis results show that a significant increase in both the p-JNK (a JNK activation form) and cleaved caspase-3 levels was observed in 5.0 mM PQ-exposed fly brain tissues, while similarly exposed flies supplemented with calycosin (100 µM) showed a reduction in the levels of p-JNK and caspase-3 (Figure 5A). A similar trend for cleaved caspase-3 activity was observed in the fly brain samples exposed to PQ and PQ + calycosin after immunostaining and caspase-3 and -9 biochemical activity assays (Figure 5B–D). Overall, these results show that calycosin supplements alleviate PQ-induced neurodegeneration via suppressing JNK and caspase-3 activation, which is responsible for DA neuronal cell death in exposed organisms.

### 3.5. Calycosin Supplementation Improves Mitochondrial Functions in PQ-Exposed Canton S Files

**Figure 6:**
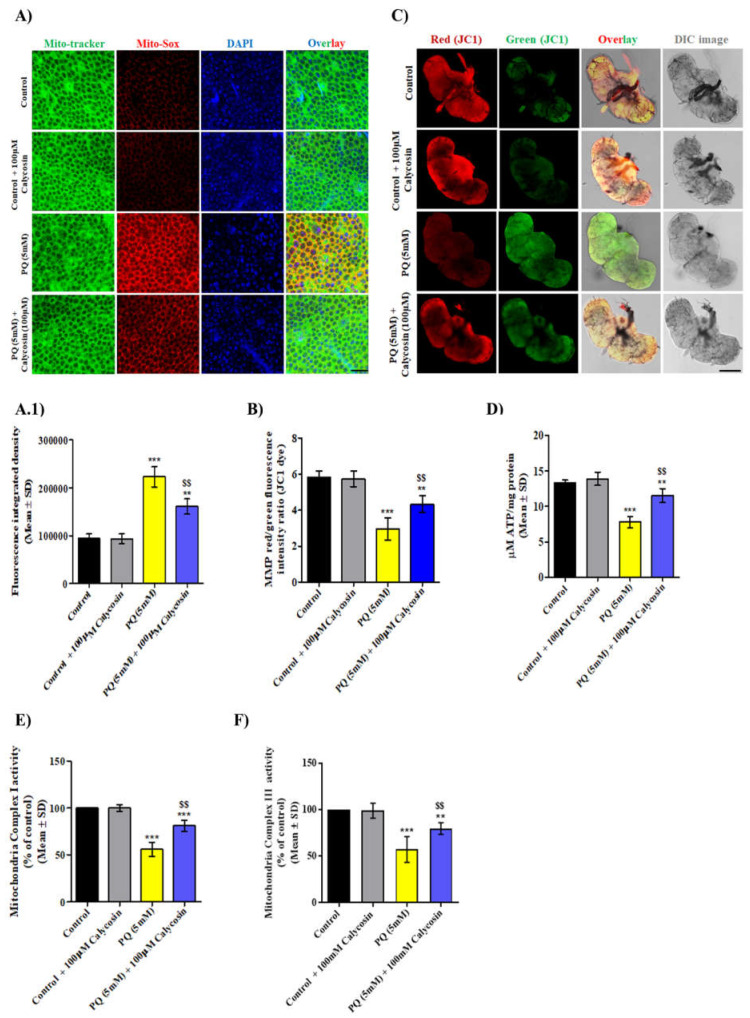
Calycosin administration improves PQ-induced mitochondrial functions in exposed flies. (**A**) Representative confocal microscopic images showing Mito-SOX O_2_**^−^.** staining in the live brain tissues from the control, 5 mM PQ and with PQ (5 mM) + 100 µM calycosin-exposed flies and (**A.1**) graphs showing Mito-SOX O_2_**^−^.** fluorescence integrated density in the control and exposed flies. Scale bars—20 µm. Representative graph (**B**) and confocal images (**C**) showing mitochondrial membrane potential (in terms of red to green fluorescence intensity measurement) using the JC-10 dye assays in the brain samples of control, 5 mM PQ and with PQ (5 mM) + 100 µM calycosin-exposed flies after 48 h. Scale bars—100 µm. Graphs showing ATP level (**D**), mitochondria complex-I (**E**) and -II (**F**) activity in the brain samples of the control, 5 mM PQ and with PQ (5 mM) + 100 µM calycosin-exposed flies after 48 h. Values represent mean ± SD (*n* = 3). Significance ascribed as ** = *p* < 0.01 and *** = *p* < 0.001 vs. control and ^$$^ = *p* < 0.05 vs. PQ (5 mM) exposure. PQ, paraquat; SD, standard deviation.

Earlier studies reported that PQ-induced higher OS levels and mitochondrial dysfunction are prominent early features initiating further apoptosis, leading to the death of DA neuron cells, thereby inducing Parkinson’s-like phenotypes [56,57]. Higher mitochondrial ROS production (O_2_**^−^.** generation), the loss of mitochondrial complex activities and ATP levels and mitochondrial membrane potential (MMP) are the most common characteristics of mitochondrial dysfunction in neurotoxicant-exposed organisms. Therefore, we first observed the mitochondrial O_2_**^−^.** level using Mito-SOX staining in PQ- and PQ with calycosin-exposed flies. Consistent with the results of earlier studies, a significantly increased O_2_**^−^.** level was found in the brain tissues of PQ-exposed flies compared with control flies. Conversely, calycosin supplementation shows a significantly reduced O_2_**^−^.** level in a similarly exposed flies compared to PQ-exposed flies (Figure 6A). We next investigated whether calycosin could restore the MMP in the neuron cells of PQ-exposed flies. We used JC-1 dye live staining to measure MMP. PQ-exposed flies exhibited a much lesser MMP (in terms of the decreased red to green fluorescence intensity) then control flies, whereas calycosin supplementation significantly restored MMP in similarly exposed flies (Figure 6B,C). In addition, calycosin supplements administered to PQ-exposed flies significantly recovered the mitochondrial complex I and III activity and ATP levels (Figure 6D–F). These results suggest that calycosin supplementation attenuates mitochondrial dysfunction in PQ-exposed flies, thus protecting the neuron cells from PQ-induced oxidative damage and apoptotic cell death.

### 3.6. Calycosin Administration Confers Protection against PQ-Induced Neurotoxicity Partly via a Mechanism Involving Autophagy Response

**Figure 7:**
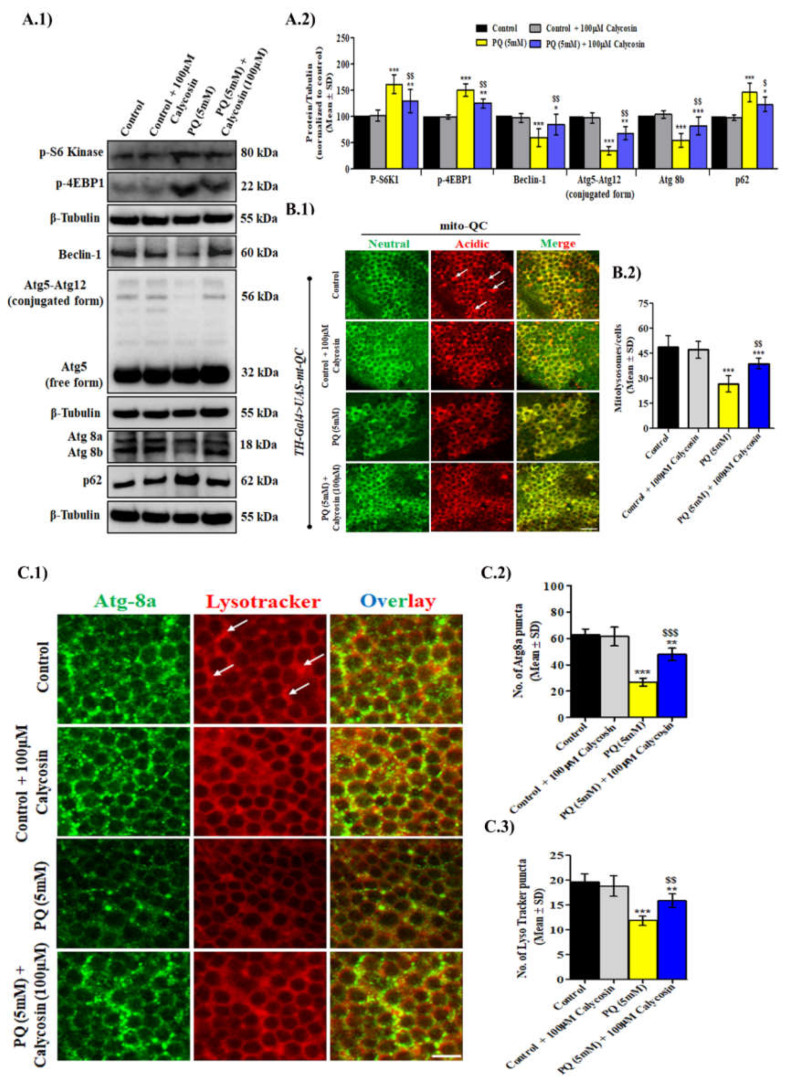
Calycosin confers protection against PQ-induced neurotoxicity through the modulation of the autophagy response in exposed flies. Western blot images (**A.1**) and their densitometry analysis graph (**A.2**) showing p-S6K1, p-4EBP1, beclin-1, Atg5-Atg12, Atg8b, and p62 in protein samples from the brains of control, 5 mM PQ and with PQ (5 mM) + 100 µM calycosin-exposed flies after 48 h. Values represent mean ± SD (*n* = 3). Significance ascribed as * = *p* < 0.05, ** = *p* < 0.01 and *** = *p* < 0.001 vs. control and ^$^ = *p* < 0.05 and ^$$^ = *p* < 0.01 vs. PQ (5 mM) exposure. (**B.1**) Representative confocal images showing mitophagy using mito-QC staining in the brain samples of control, 5 mM PQ and with PQ (5 mM) + 100 µM calycosin-exposed *TH-Gal4/UAS-mito-QC* flies after 48 h and (**B.2**) quantification showing mitolysosomes in control and exposed flies. Confocal images (**C.1**) showing Atg8a (green) and Lysotracker staining (red) of the brain tissues in the control, 5 mM PQ and with PQ (5 mM) + 100 µM calycosin-exposed flies after 48 h and quantification showing Atg8a (**C.2**) -and Lysotracker -puncta (**C.3**) in control and exposed flies. Values represent mean ± SD (*n* = 3). Significance ascribed as ** = *p* < 0.01 and *** = *p* < 0.001 vs. control and ^$$^ = *p* < 0.05 and ^$$$^ = *p* < 0.001 vs. PQ (5 mM) exposure. PQ, paraquat; SD, standard deviation. Scale bars—20 µm.

As calycosin supplementation protects neurons cells from PQ-induced oxidative damage, we further attempted to examine whether calycosin administration activates the autophagy response pathway in PQ-exposed flies. Autophagy is a lysosome-driven process to get rid of damaged cellular organelles, protein aggregates and toxic cargos that can lead to cellular dysfunction. Dysregulated or defective autophagy has been recognized as a key pathogenic process in most NDs, including PD. Numerous in vitro and in vivo studies have shown that flavonoids exert protective roles on PQ-induced neurotoxicity and have highlighted their neuroprotective efficacy via autophagy activation [22,24,25]. To test whether calycosin regulates autophagy, we examined the levels of the main proteins of autophagy regulation (beclin, Atg5-Atg12 (involved in Atg8 lipidation and autophagosome vesicle membrane expansion), Atg8a/8b and Ref(2)p (a human homologue of p62 protein)) in flies’ brain tissues using Western blot assays. The levels of these proteins were found to be significantly (*p* < 0.01) decreased in PQ-exposed flies compared with control flies, whereas they were significantly (*p* < 0.01) increased in PQ + calycosin-exposed flies as compared with the PQ alone-exposed flies under similar experimental conditions (Figure 7A). Conversely, the level of p62, an essential mitophagy regulator that indicates the damaged mitochondria’s accumulation, was observed to be dramatically increased in PQ-exposed flies compared with control flies, while a significant decrease was found in PQ + calycosin-exposed flies (Figure 7A). Afterwards, we examined the mitophagy level in the *TH-Gal4/UAS-mito-QC* flies’ neuron cells using the mito-QC probe staining assay. The PQ-exposed flies showed a lower number of neuron cells with mitolysosomes than control flies, whereas PQ + calycosin-exposed flies exhibited a higher number of neuron cells with mitolysosomes (Figure 7B). Furthermore, to confirm the role of calycosin in autophagy stimulation, immunostaining of the adult flies brain tissues was also carried out with Lyso-Tracker Red, a vital dye used for the detection of acidic organelles, lysosome, and Atg8a for the detection of autophagosome complex [58]. Decreased autophagolysosome formation was observed in PQ-exposed flies as compared to control flies, while the reverse was found in PQ + calycosin-exposed flies under similar experimental conditions (in magnified images, autophagolysosomes were represented by the yellow color due to the merger of atg8a (green) and Lyso Tracker (red) dye) (Figure 7C). Collectively, the results suggest that calycosin elicits neuroprotection against PQ toxicity through the regulation of autophagy signaling pathways involving mitolysosomes and autophagosomes.

Additionally, we assess whether the mTOR signaling pathway is involved in the autophagy regulation in PQ- and PQ+ calycosin-exposed flies. The Western blot results show that exposure to PQ resulted in a significant increase in the level of p-S6K and 4EBP1 (downstream targets of mTOR signaling), whereas this effect was significantly abolished by calycosin administration in PQ-exposed flies (Figure 7A). This result indicates that mTOR signaling pathways were activated by PQ exposure, and negative regulation of autophagy stimulation resulted in the aggravation of PQ-induced neurotoxicity.

## 4. Discussion

In the present in vivo study, we addressed the protective efficacy of Calycosin on PQ-induced neurodegeneration in exposed *Drosophila*.

Paraquat (PQ) is commonly known as a cellular redox–cycling agent, and exerts its toxicity via the excessive generation of ROS. The accumulation of ROS and the diminution of reducing agents leads to higher OS, and the resulting damage to the lipids, protein and DNA potentially causes apoptotic cell death [59]. Notably, we observed significantly higher O_2_^•−^ generation along with reduced SOD activity in the brains of PQ-exposed *Canton S* flies after 48 h compared to control flies, while the reverse was observed in flies fed with calycosin under a similar exposure regimen. Furthermore, decreased SOD activity and higher O_2_^•−^ generation may lead to the formation of other reactive species, especially peroxynitrite anion ONOO^−^, in exposed organism. A significantly lower accumulation of ONOO^−^ anions was observed in the brains of calycosin fed PQ-exposed flies than PQ-exposed flies may be due to increased SOD activity, which makes them further resistant to PQ-induced toxic insults. This observation finds support from earlier studies, wherein SOD or its mimetic provided protection against PQ-induced damage by maintaining the cellular antioxidant defense system [60,61]. Since lipid peroxidation (LPO) is also considered as one of the major mechanisms of PQ-induced toxicity, a lower level of LPO was observed in the brains of PQ-exposed flies fed with calycosin as compared to in PQ-exposed flies. Furthermore, the resistive effect of calycosin against PQ-mediated OS is also evidenced by the lesser depletion of GSH content, a major component of the overall antioxidant defense of cells, in calycosin-fed PQ-exposed flies. This finding suggests that the protective effect of calycosin could be attributed to its antioxidant properties.

The overproduction of ROS and OS, leading to the DA neurons loss in environmental toxicant-induced PD models, has been widely reported [11,12,62]. In PD, the dopamine depletion due to either/both DA neuron loss or dopamine oxidation is traditionally associated with cardinal motor symptoms such as rigidity, bradykinesia and tremors [63]. We suggest that calycosin was able to inhibit DA neuron loss and dopamine reduction, showing evidence of a potential neuroprotective compound, which we trust has a significant correlation with the improvement in motor performance in PQ-exposed flies. This is further consistent with prior studies, which indicate the significant correlation between motor dysfunction and dopamine depletion. Further, quercetin, curcumin, caffeic, vanillic, squalene, coumaric acid, ferulic acid, and *Decalepis hamiltonii* root extract, *B. glabra*, *Sanguisorba officinalis, Bacopa monnieri,* and *Zedoariane rizhoma* extracts showed protective potential against PQ-induced mortality, DA neuron loss, locomotor impairment and oxidative damage in different models [24,64,65,66,67,68]. Recently, calycosin alleviated MPTP-induced PD-like symptoms in a mouse model by improving DA neurons’ survival and locomotor functions [40].

In addition, PQ-induced OS has been implicated in the neuronal cell death through the JNK phosphorylation and caspase-3 activation. We found a significant increase in JNK phosphorylation and cleaved caspase-3 level in PQ-exposed flies after 48 h, while significantly reduce in calycosin-fed PQ-exposed flies under similar exposure regimen. Our results indicates that lower OS (increased SOD activity, decreased ROS/RNS active species production and MDA content, and lesser depletion of GSH content) in calycosin-fed PQ-exposed flies would suppress JNK-caspase-3-mediated apoptotic cell death. This observations support from earlier study of Shukla et al. (11), where they demonstrated JNK reduction using knockdown of JNK (bsk) in PQ-exposed flies provide protection to neuronal cells from their degeneration, resulted in improved flies survival than similarly exposed *w^1118^* flies and confirmed the role of JNK in PQ-induced neurodegeneration in exposed organism. Thus, our observation of reduced neuronal cell death due to lower JNK level in calycosin fed PQ-exposed flies strengthens the above. However, further studies would be conducts to confirm that calycosin mediated rescuing of PQ-induced neuronal cell death and degeneration by inhibiting of JNK, using of JNK mutant flies. Earlier studies also showing that plant extract/flavonoids molecules may function as specific inhibitors of JNK and caspase-3 activity in several experimental PD models [40,69,70,71,72,73].

Emerging evidence has shown that mitochondrial dysfunction plays an essential role in the development and progression of several NDs, including PD [74,75]. Post-mortem studies of brains of PD patients reported that higher levels of mitochondrial dysfunction are closely linked with the degeneration of neurons, and are associated with PD pathogenesis [76,77]. In *Drosophila*, *PINK1^B9^* mutation leads to the mitochondrial dysfunction, which is closely linked with the age-related degeneration of muscles and neurons, and is associated with a shortened health- and lifespan [78,79]. Given the critical association of mitochondrial dysfunction with the PD development and progression, it is necessary to find the potential novel medicines that can stimulate mitochondrial defense signaling pathways in NDs’ pathogenesis. Our result show that calycosin administration not only significantly lowers mitochondrial O_2_^•−^ generation but also enhances the ATP level, mitochondrial membrane potential (MMP) and complex-I and -III enzyme activities to resist OS induced by PQ in exposed flies. These results indicate that the neuroprotective function of calycosin might be involved in the maintenance of mitochondrial homeostasis and functions, as well as providing protection against elevated ROS production in the mitochondria. Consistent with earlier studies showing the selective activation of UPR^mt^, the important protective mitochondrial stress signaling pathway by GTP supplements significantly attenuated mitochondrial dysfunction (lowered ATP level, MMP and mitochondria respiration activity, while the increased ROS level) in the *Drosophila PINK1^B9^* mutation and PQ and rotenone-exposed organisms [74]. Thus, further research is also warranted to investigate whether calycosin supplements have a regulatory effect on UPR^mt^.

As OS characterizes PQ-induced PD conditions, a growing body of evidence has reported that the impairment of autophagy elevates the toxicity caused by PQ, while its stimulation reverses this effect in several experimental in vitro and in vivo studies [14,15,17,24,26]. Earlier in vitro studies stated that natural compounds, i.e., resveratrol, curcumin and vasicinone, activate autophagy, promoting the degradation of accumulated α-synuclein aggregates induced by PQ in human neuronal culture cells [24,26,80]. Likewise, other studies also reported that autophagy stimulation by rapamycin exerted a protective effect against OS (in terms of increased MDA content) and apoptotic cell death induced by neurotoxicants in both the in vitro and in vivo PD models [25,81]. In the same way, we observed that calycosin also stimulates autophagy and promotes the clearance of toxic cargos and the damaged organelles from the cells induced by PQ in exposed organisms. The expression of various autophagy-related proteins was observed to be significantly decrease, except the p62 level, which was found to be increased, in the PQ-exposed flies after 48 h compared to the control flies, whereas the reverse was found in the calycosin-fed PQ-exposed flies. Lu et al. [82] showed that calycosin has a beneficial role against doxorubicin-induced cardiotoxicity through autophagy stimulation in zebrafish models, which supports our present study. Furthermore, autophagy activation [83,84] leads to a lowered OS and finally to reduced apoptotic cell death, indicating that there is a crosstalk between autophagy and apoptosis. Therefore, a reduced OS level may increase the stress adaptive response in organisms resistant to xenobiotics stress, indicating the protective effect of autophagy against OS-mediated insults. Further, we examined whether calycosin has effects on mTOR-dependent and independent pathways in the regulation of autophagy to the amelioration of PQ-induced neurotoxicity. The mTOR serves a critical function in cellular growth and is reported as a negative regulator of autophagy via modulating the energy balance between cells’ metabolic pathways. We found higher levels of phosphorylated -S6K and -4EBP1, downstream targets of mTOR complex-I, in PQ-exposed flies after 48 h, suggestive of a poor energy balance between the anabolic and catabolic pathways, resulting in autophagy inhibition and apoptosis induction. Meanwhile, calycosin supplementation exhibited decreased phosphorylation levels of S6K and 4EBP1 in exposed flies, which might indicate a maintained energy balance between anabolic and catabolic processes. This finding is also consistent with a prior study wherein decreased levels of pS6K/p4EBP1 resulted in reduced apoptosis as well as neurotoxicity in rat cerebral cortex tissues exposed with methylmercury [83]. Thus, autophagy stimulators might be suitable applicants for interventions in NDs.

## 5. Conclusions

**Figure 8:**
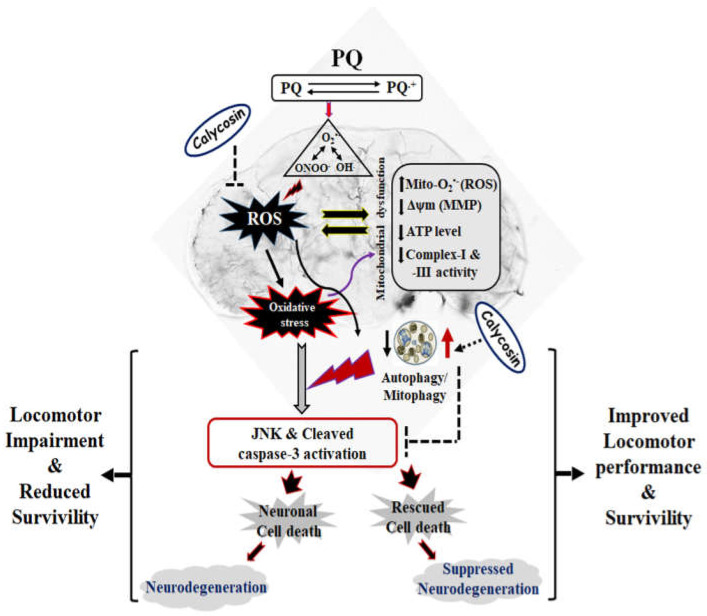
Schematic representation of the protective efficacy of calycosin administration against neuronal cell death, and locomotor impairment and reduced survival of *Drosophila* after exposure to PQ. Schematic model showing a neuroprotective potential of calycosin on PQ-induced PD-like phenotypes by improving the DA neuronal health, better locomotor performance, and increasing fly survival, primarily governed by lower reactive species (O_2_^•−^/ONOO^−^) formation, pJNK-caspase-3-mediated dopaminergic neuronal cell death and improving mitochondrial functions and restoring autophagy in exposed organisms.

Collectively, the current study shows a neuroprotective potential of calycosin on PQ-induced PD-like phenotypes using *Drosophila* as a model organism, as evidenced by improved DA neuronal health, locomotor performance, and increased fly survival, primarily governed by lower ROS (O_2_^•−^/ONOO^−^) formation, pJNK-caspase-3 mediated neuronal cell death, and improving mitochondrial functions and restoring autophagy in exposed organism (Figure 8). Although, autophagy stimulators might be suitable applicants for the interventions of NDs, it is tempting to speculate that investigating the mechanistic interrelation between autophagy and cellular redox signaling would be promising for further studies to shed light on the challenges of the advancement of novel neuroprotective therapeutic approaches.

## Figures and Tables

**Table 1:** List of primary antibodies and their dilutions used for Western blotting in the present study.

Antibodies	Host	Dilution	Catalog Number	Company
TH	rabbit	1:1000	#AB-152	Sigma Aldrich (Merck Millipore), Darmstadt, Germany)
pJNK	rabbit	1:1500	#9661	Cell Signaling Tech. (Danvers, MA, USA)
JNK	rabbit	1:1000	#SC-571	Santa Cruz Biotech. (Dallas, TX, USA)
cleaved caspase-3	rabbit	1:1000	#9661	Cell Signaling Tech. (Danvers, MA, USA)
p-S6Kinase	rabbit	1:1000	#9209	Cell Signaling Tech. (Danvers, MA, USA)
p-4EBP1	rabbit	1:1000	#2855	Cell Signaling Tech. (Danvers, MA, USA)
beclin-1	rabbit	1:500	#OSB00021W	Osenses
Atg5	rabbit	1:1000	# NB110-53818	Novus Biologicals (Centennial, CO, USA)
Atg8a/8b	mouse	1:500	#A5441	Sigma Aldrich (Merck Millipore), Darmstadt, Germany)
p62	rabbit	1:1000	#ab178440	Abcam (Cambridge, UK)
DNPH	rabbit	1:5000	#ab178440	Abcam (Cambridge, UK)
β-tubulin	mouse	1:1500	#E7	Developmental Studies Hybridoma Bank (Iowa City, IA, USA)

## Data Availability

Data is contained within the article or Supplementary Material.

## Supplementary Materials

The following supporting information can be downloaded at: https://www.mdpi.com/article/10.3390/antiox11020222/s1, Figure S1. The protective effect of Calycosin supplements on survival of flies pre-treated with PQ. Survival assays were performed by using 3–5-day-old adult male flies that were fed with a different concentration of Calycosin (100.0 μM, 200.0 μM and 400.0 μM) after 24 h, 48 h, and 72 h post PQ treatment in 5.0% sucrose solution on filter paper through ingestion, and the counting of surviving flies was performed every 24 h post-exposure until all of the flies died, and plotted the survival percentage graphs on 24 h, 48 h, 72 h, 96 h and 120 h period. Survival data showing that Calycosin feeding at 100 μM concentration after 24h post PQ exposure yield maximum significant protection (*p* < 0.001) against PQ-induced toxicity with 46% survivality (A), while feeding at 400 μM after 48 h and 72 h post PQ exposure yield maximal survival protection (~50%) on day 5 against PQ-induced toxicity (B–C). Data are presented in at least three independent experiments with 100 flies/group. Data presented are mean ± SD (*n* = 3). Significance ascribed as $ = *p* < 0.05, $$ = *p* < 0.01 and $$$ = *p* < 0.001 vs. PQ (5mM) exposure. PQ, paraquat; SD, standard deviation.
