# Supplementary material for: Calycosin Alleviates Paraquat-Induced Neurodegeneration by Improving Mitochondrial Functions and Regulating Autophagy in a Drosophila Model of Parkinson’s Disease
Source: Antioxidants (Basel). 2022 Jan 24;11(2):222. doi: 10.3390/antiox11020222 (PMC8868496; doi:10.3390/antiox11020222)
Supplement: Supplementary file 1 [file antioxidants-11-00222-s001.zip › antioxidants-1518400-supplementary.pdf]

Calycosin alleviates paraquat-induced neurodegeneration by improving mitochondria functions and regulating autophagy in a *Drosophila* model of Parkinson's disease

Hitesh Singh Chaouhan[#1], Xin Li[#1], Kuo-Ting Sun[#2,3], I-Kuan Wang[4,5], Tung-Min Yu[6,7], Shao-Hua Yu[8], Kuen-Bao Chen[12*], Wei-Yong Lin[*9,10,11], Chi-Yuan Li[*1,12]

1. Graduate Institute of Biomedical Sciences, China Medical University, Taichung, 40402, Taiwan.
2. Department of Pediatric Dentistry, China Medical University Hospital, Taichung, 40402, Taiwan.
3. School of Dentistry, China Medical University, Taichung, 40402, Taiwan.
4. Division of Nephrology, China Medical University Hospital, Taichung, 40402, Taiwan
5. Department of Internal Medicine, School of Medicine, China Medical University, Taichung, 40402, Taiwan.
6. Division of Nephrology, Department of Internal Medicine, Taichung Veterans General Hospital, Taichung, 40402, Taiwan.
7. School of Medicine, China Medical University, Taichung, 40402, Taiwan.
8. Department of Emergency Medicine, China Medical University Hospital, Taichung, 40402, Taiwan.
9. Department of Medical Research, China Medical University Hospital, Taichung, 40402, Taiwan
10. Graduate Institute of Integrated Medicine, China Medical University, Taichung, 40402, Taiwan
11. Brain Diseases Research Center, China Medical University, Taichung, 40402, Taiwan
12. Department of Anesthesiology, China Medical University Hospital, Taichung, 40402, Taiwan

# Hitesh Singh Chaouhan, Xin Li, Kuo-Ting Sun - Joint first author.
* Kuen-Bao Chen, Wei-Yong Lin and Chi-Yuan Li - Joint corresponding author.

Correspondence:
Chi-Yuan Li, MD, MS, Graduate Institute of Biomedical Sciences, China Medical University, Taichung, 40402, Taiwan, E-mail: cyli168@gmail.com, cyli168@mail.cmu.edu.tw
ORCID ID: 0000-0003-3390-7568

Supplementary Figure

Figure S1. The protective effect of Calycosin supplements on survival of flies pre-treated with PQ. Survival assays were performed by using 3-5-day-old adult male flies that were fed with a different concentration of Calycosin (100.0 μM, 200.0 μM and 400.0 μM) after 24h, 48h, and 72h post PQ treatment in 5.0% sucrose solution on filter paper through ingestion, and the counting of surviving flies was performed every 24h post-exposure until all of the flies died, and plotted the survival percentage graphs on 24h, 48h, 72h, 96h and 120h period. Survival data showing that Calycosin feeding at 100 μM concentration after 24h post PQ exposure yield maximum significant protection ($p<0.001$) against PQ-induced toxicity with 46% survivality (Fig. S1A), while feeding at 400 μM after 48h and 72h post PQ exposure yield maximal survival protection (~50%) on day 5 against PQ-induced toxicity (Fig. S1B-C). Data are presented in at least three independent experiments with 100 flies/group. Data presented are mean ± SD (n=3). Significance ascribed as \$=p<0.05, \$\$=p<0.01 and \$\$\$=p<0.001 vs. PQ (5mM) exposure. PQ, paraquat; SD, standard deviation.

A)

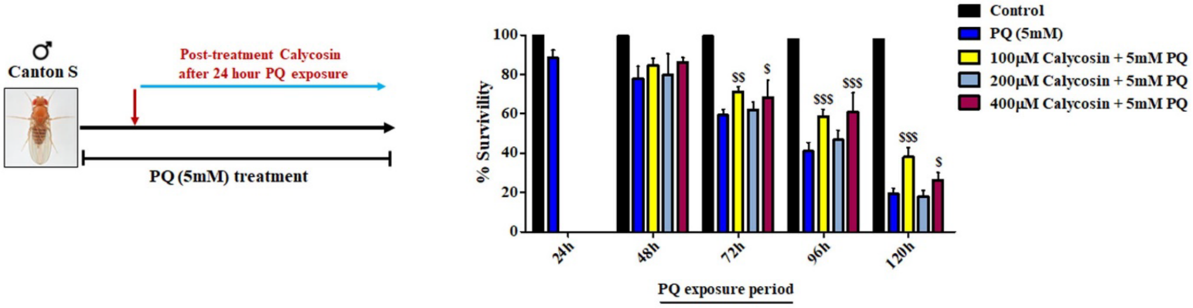

♂
Canton S
Post-treatment Calycosin
after 24 hour PQ exposure
PQ (5mM) treatment
% Survivility
PQ exposure period
24h
48h
72h
96h
120h
Control
PQ (5mM)
100µM Calycosin + 5mM PQ
200µM Calycosin + 5mM PQ
400µM Calycosin + 5mM PQ

B)

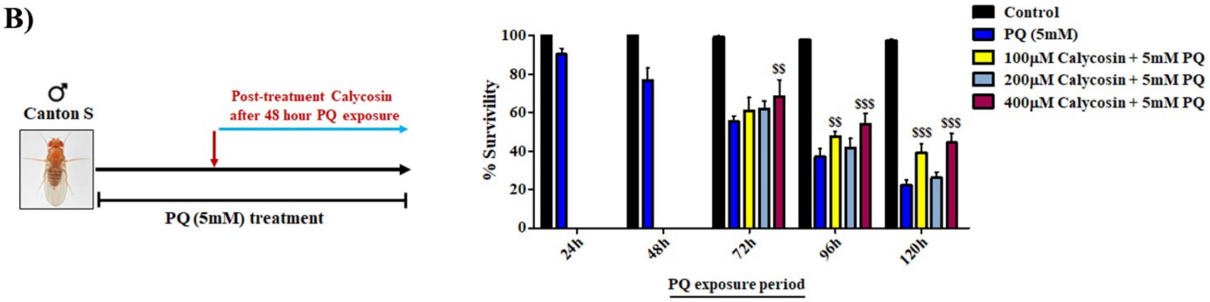

♂
Canton S
Post-treatment Calycosin
after 48 hour PQ exposure
PQ (5mM) treatment
% Survivility
PQ exposure period
24h
48h
72h
96h
120h
Control
PQ (5mM)
100µM Calycosin + 5mM PQ
200µM Calycosin + 5mM PQ
400µM Calycosin + 5mM PQ

C)

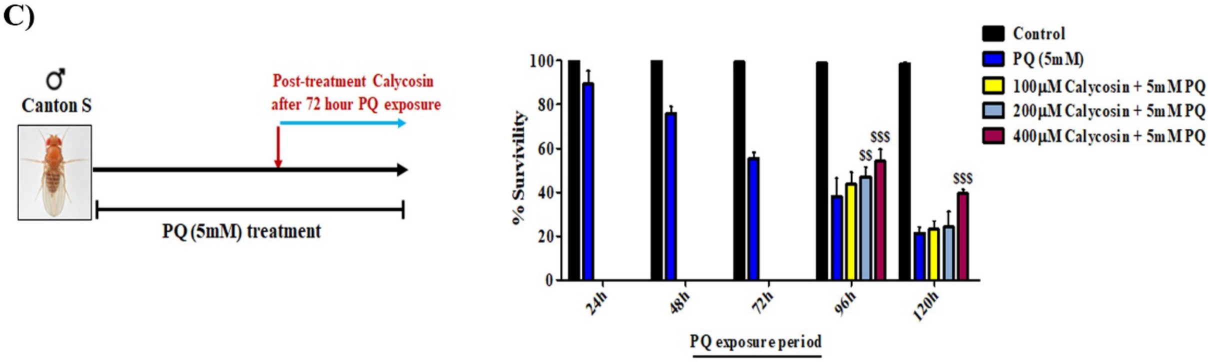

♂
Canton S
Post-treatment Calycosin
after 72 hour PQ exposure
PQ (5mM) treatment
% Survivility
PQ exposure period
24h
48h
72h
96h
120h
Control
PQ (5mM)
100µM Calycosin + 5mM PQ
200µM Calycosin + 5mM PQ
400µM Calycosin + 5mM PQ
